# Practical Notes and Formulæ

**Published:** 1878-08-20

**Authors:** 


### *PRACTICAL NOTES AND FORMULÆ.*

### TYPHO-MALARIAL FEVER.

The following note from one of our esteemed readers I hope will attract attention. The subject is an important one, and, we trust, will call forth a practical contribution from one or more of our readers. The editors have nothing specially new or important on the subject:

ELMO, TEXAS, July 11, 1878.

*Dear Sir—*

Could you not get up an exhaustive paper on typho-malarial fever? I have as yet seen scarcely anything on the subject Even the text-books barely mention it. Yet, it is very common here, and is very fatal. I should like to have the pathological leisons—especially of the bowels. I saw some fifteen cases of it in Terrell last fall. There have already been some four or five here within the last three or four months, and this season bids fair to breed a large crop of it.

You could not get out a more timely and useful paper.

About fifty per cent. died of it.

Yours, truly,
R. FOWLER, M.D.

### RIGID OS—CAUTION.

Dr. Evans (in *Med. Brief*) recommends the following large opiate as very efficient in protracted labors caused by rigid os.

| | |
|---|---|
| R.—Opium | 1 grain. |
| Morphine | $\frac{1}{8}$ of grain. |
| Subnit of bismuth | 10 grains. |

He says we get in this combination "the prompt effect in the morphine and the more lasting and uniform effect by combining the two." We approve of the method of a decided opiate in such cases, but the practitioner must look out for idiosyncrasies as to opium, and remember that so large a dose as above advised would not be borne with safety in every case.

In the same journal, Dr. Ruff advises for tedious labors—

| | |
|---|---|
| Morphiæ sulph | 1 grain. |
| Chloral hydrate | 30 grains. |
| Tinct. gelseminum | $1\frac{1}{2}$ drachms. |
| Aquæ to make | 2 ounces. |

Dose, tablespoonful every half hour. Here again we must caution the practitioner that the dose of gelseminum is too large for indiscriminate use.

### SALICYLIC ACID.

*Editor Record—*

Will you please tell me, through your journal, what the best vehicle is for giving salicylic acid, and oblige

W. C. R.

To the above inquiry, we reply that on page 156 of our last year's volume may be found an article on this subject, in which Dr. Barkley, of Kentucky, suggests the use of sweet spirits of nitre as the best solvent for salicylic acid.

| | |
|---|---|
| R.—Salicylic acid | dr. j. |
| Sweet spts. nitre | dr. iv. |

This may be diluted with water and given in doses of two to four drachms for an adult. One ounce of spirits nitre will dissolve 16 grains of the pure acid. The remedy may be used in the form of the salicylate of soda. The following formulæ have been recommended:

| | |
|---|---|
| R.—Acidi salicylici | dr. j. |
| Olei amygdalæ (expr.) | dr. v. |
| Pulv. accaciæ | dr. ijss. |
| Syrupi amygdalæ | dr. vj. |
| Aquæ aurantii flores | oz. ijj. |

M.

### FOR CHILDREN (NO. 1).

| | |
|---|---|
| R.—Acidi salicylici | dr. j. |
| Spts. rect | dr. ijss. |

Dissolve.

(NO. 2.)

Potassii citratis........................dr. j.
Syrupi aurantii........................dr. ij.
Aquæ............................dr. iijss.

Mix with No. 1 and filter, and then dilute with water to taste. Dose, one teaspoonful.

TO PREVENT DIPHTHERIA.

Chapman, in his work on the "Antagonism of Alcohol to Diphtheria," states that those exposed to infection should be protected by having a free circulation of air through the house, and by taking a certain amount of alcohol each day. The following formula is recommended:

R.—Quinoidine, } aa................gr. xij.

Cinchoniæ sulph } to................xxiv.

Acid. sulph. aromat....................dr. ij.

Sp. Frumenti........................oz. viij.

M. S. Fifteen drops to a tablespoonful four or five times a day.

The same formula, with quinia substituted for quinoidine, is given to a patient who has the disease, only shortening the interval to one or two hours. He holds also that alcohol is antidotal and remedial to scarlatina. In the latter disease, he uses—

R.—Liq. ammoniæ acetat.,
Sp. frumenti......................aa. oz. ij.

M. S. Two teaspoonsful every hour and a half in water.

FOR NERVOUS PALPITATIONS.

R.—Pulv. assafœtida................grs. xxxvj.
Digitalis pulv........................grs. iij.
Ext. valerianæ......................grs. vij.

M. and make 20 pills, one to be taken morning and evening. NAPHEY.

SUBSTITUTE FOR MOTHER S MILK.

Dr. Marsh (in Ob. Gazette) mentions the following as the nearest approximation to the mother's milk that can be made:

Cow's milk, fresh...................... 3 parts.
Water................................ 1 part.

Sugar of milk one teaspoonful to a half pint of the mixture.

FOR COLLEQUITIVE DIARRHŒA.

A favorite prescription of Prof. DaCosta, of Philadelphia, in the latter stages of consumption attended by night sweats, diarrhœa and debilitating cough, is the following:

R.—Morphæ acetatis.................... grs. ij.
Potassii cyanidi...................... grs. j.
Acidi acetici.......................... dr. j.
Ext. pruni. verginianæ fluidi
Misturæ accaciæ, aa................ oz. ij.

M. S. A teaspoonful 4 to 6 times per day.

Another good formula for this condition is the following:

R.—Muriated tinc. iron.................. oz. j.

Muriatic acid, dilute................ dr. ij.

M. Twenty drops before meals in half teacupful of sweetened water, drawn through a quill to proteet the teeth.

ORGANIC HEART DISEASE.

In palpitation from valvular disease or other organic heart trouble, the following is very useful:

R.—Spts. nitre compositus.............. dr. ij.
Tinc. hyosciami.................... dr. jss.
Decocti senegæ...... .............. oz. iij.
Mesturæ camph...................... oz. iv.

M. Dose, one or two tablespoonfuls frequently taken.

Another—

R.—Potassii bromidi...... ...... gr. xv.

Tinc. digitalis.............. ........ gtt. v.

To be taken two or three times per day.—*Ib.*

COUGH MIXTURE.

In any severe cough where the tongue is red or the throat sore, the following is recommended by Dr. Powell, of Brompton, as a superior remedy:

R.—Potassii chloratis...... .......... gr. xl.
Morph. muriatis.......... .......... grs. ij.
Glycerin.......................... oz. ½.
Syrup... ........................ oz. 3½

M. To be taken undiluted and slowly for both its local and constitutional effect. Dose, one teaspoonful three or four times a day.

### LEUCORHŒA.

Dr. Cronyn (in Buffalo Medical Journal) recommends the following as injection in ulceration of the os to be varied in strength according to the severity of the case:

Acidi carbolici,
Liq. plumbi subacet,
Fluid Ext. papaveris,
Glycerine,
Aqua rosa.

And also the following tonic medicines, by the stomach, for the relief of the dozen and one complaints always attendant upon such cases:

| | |
|---|---|
| Tinct. ferri mur | dr. iii. |
| Tinct. nucis vomica | dr. ii. |
| Tinct. calumbo | oz. ii. |
| Aqua cinnamoni | oz. ii. |
| Syrupi simplicis | q. s. |
| Ft. mist | oz. viii. |

Signa.—One tablespoonful three time a day.

### NERVINE AND ANTISPASMODIC.

A favorite prescription in the Hospital of Chest Diseases, London, is the following, useful in epilepsy, chorea, dysmenorrhæ, hysterica epileptica and like nervous conditions:

| | |
|---|---|
| R.—Potassii bromidi | gr. x. |
| Tinc. conii | gtt. xxx. |
| Tinc. val. ammoniæ | gtt. xx. |
| Aquæ camph | oz. j. |

For one dose thrice daily.

### LACTOPEPTINE.

In the treatment of infantile diarrhœa, produced by imperfect digestion, we have had most satisfactory results from the use of lactopeptine; also, in cases of impaired digestion in old persons. This is one of the most valuable pharmaceutical preparations that has been placed in the hands of the profession. We take pleasure in attesting to its value from a considerable experience in the use of it.—*Cin. Lan. and Ob.*

### TONIC IN PHTHISIS.

Dr. Curran, of Dublin, uses the following as a superior tonic in the latter stages of consumption:

| | |
|---|---|
| R.—Zinci oxidi | gr. ij. |
| Ext. conii | gr. j. |

To be taken in pill three times a day.

### CASTOR OIL EMULSION.

Dr. Ezell (in the Louisville Medical News) recommends the following mixture as serving to deprive the oil of every disagreeable feature. It would seem to be well adapted as a purgative for delicate stomachs, where castor oil is indicated. In children especially it would answer well, and as a laxative in childbed—

| | |
|---|---|
| R.—Oleum ricini | 1 ounce. |
| Tinc. cardamon com | ½ " |
| Ol. gaultheriæ | 4 drops. |
| Pulv. accaciæ, Pulv. sacch alb. } aa | 2 drachms. |

Cinnamon water to make 4 ounces. Mix.

He does not direct the dose. We suggest that for an adult one-half the mixture should be given, and repeated in two hours. For a child one or two years old a tablespoonful every hour until it operates. The cinnamon being astringent, we suggest mint water instead.

### INSECTS.

The *Journal of Chemistry* says that hot alum water is the best insect destroyer known. Put the alum into hot water and let it boil till the alum is dissolved; then ayply it hot with a brush to all cracks, closets, bedsteads, and other places where any insects are found. Ants, bed-bugs, cockroaches and creeping things are killed by it; while there is no danger of poisoning the family or injuring property.

### SUMMER COMPLAINT.

Dr. Gibbon, of North Carolina, claims great success in summer complaint of children. He gives 10 grains of sub. nit. bismuth every 1, 2 or 3 hours according to severity of the case. In cholera infantum he gives first a dose of calomel and follows with the above treatment.

### CINNAMON IN HEMORRHAGE.

| | |
|---|---|
| R.—Oil cin | 1 ounce. |
| Alcohol (98°) | 8 " |

Dose, 5 to 30 drops oft repeated.

